# A Novel Generation of Tailored Antimicrobial Drugs Based on Recombinant Multidomain Proteins

**DOI:** 10.3390/pharmaceutics15041068

**Published:** 2023-03-26

**Authors:** Adrià López-Cano, Neus Ferrer-Miralles, Julieta Sánchez, Jose Vicente Carratalá, Xavier Rodriguez Rodriguez, Imma Ratera, Judith Guasch, Oscar Q. Pich, Paula Bierge, Cristina Garcia-de-la-Maria, Jose M. Miro, Elena Garcia-Fruitós, Anna Arís

**Affiliations:** 1 Department of Ruminant Production, Institute of Agriculture and Food Research (IRTA), Caldes de Montbui, 08140 Barcelona, Spain; adrialopezcano@gmail.com (A.L.-C.); elena.garcia@irta.cat (E.G.-F.); 2 Institute for Biotechnology and Biomedicine, Autonomous University of Barcelona, Bellaterra, 08193 Barcelona, Spain; neus.ferrer@uab.cat (N.F.-M.); jsanchezqa@gmail.com (J.S.); josevicente.carratala@uab.cat (J.V.C.); 3 Department of Genetics and Microbiology, Autonomous University of Barcelona, Bellaterra, 08193 Barcelona, Spain; 4 Bioengineering, Biomaterials and Nanomedicine Networking Biomedical Research Centre (CIBER-BBN), Bellaterra, 08193 Barcelona, Spain; xrodriguez2@icmab.es (X.R.R.); iratera@icmab.es (I.R.); jguasch@icmab.es (J.G.); 5 Institute of Materials Science of Barcelona (ICMAB-CSIC), Campus UAB, Bellaterra, 08193 Barcelona, Spain; 6 Dynamic Biomimetics for Cancer Immunotherapy, Max Planck Partner Group, Institute of Materials Science of Barcelona (ICMAB-CSIC), Campus UAB, Bellaterra, 08193 Barcelona, Spain; 7 Laboratori de Recerca en Microbiologia i Malalties Infeccioses, Parc Taulí Hospital Universitari, Institut d’Investigació i Innovació Parc Taulí (I3PT-CERCA), Universitat Autònoma de Barcelona, 08208 Sabadell, Spain; oquijada@tauli.cat (O.Q.P.); pbierge@tauli.cat (P.B.); 8 Infectious Diseases Service, Hospital Clinic-IDIBAPS, University of Barcelona, 08007 Barcelona, Spain; cgarciad@clinic.cat (C.G.-d.-l.-M.); josemaria@miromoreno.org (J.M.M.); 9 CIBERINFEC, Instituto de Salud Carlos III, 28029 Madrid, Spain

**Keywords:** host defense peptides, antimicrobial, recombinant, nosocomial infections

## Abstract

Antibiotic resistance has exponentially increased during the last years. It is necessary to develop new antimicrobial drugs to prevent and treat infectious diseases caused by multidrug- or extensively-drug resistant (MDR/XDR)-bacteria. Host Defense Peptides (HDPs) have a versatile role, acting as antimicrobial peptides and regulators of several innate immunity functions. The results shown by previous studies using synthetic HDPs are only the tip of the iceberg, since the synergistic potential of HDPs and their production as recombinant proteins are fields practically unexplored. The present study aims to move a step forward through the development of a new generation of tailored antimicrobials, using a rational design of recombinant multidomain proteins based on HDPs. This strategy is based on a two-phase process, starting with the construction of the first generation molecules using single HDPs and further selecting those HDPs with higher bactericidal efficiencies to be combined in the second generation of broad-spectrum antimicrobials. As a proof of concept, we have designed three new antimicrobials, named D5L37βD3, D5L37D5L37 and D5LAL37βD3. After an in-depth exploration, we found D5L37D5L37 to be the most promising one, since it was equally effective against four relevant pathogens in healthcare-associated infections, such as methicillin-susceptible (MSSA) and methicillin-resistant (MRSA) *Staphylococcus aureus*, methicillin-resistant *Staphylococcus epidermidis* (MRSE) and MDR *Pseudomonas aeruginosa*, being MRSA, MRSE and *P. aeruginosa* MDR strains. The low MIC values and versatile activity against planktonic and biofilm forms reinforce the use of this platform to isolate and produce unlimited HDP combinations as new antimicrobial drugs by effective means.

## 1. Introduction

The discovery of antibiotics led to a golden age in human healthcare, providing a wide range of therapies to cope with bacterial infections [1,2]. As a result of the prevalent use, and sometimes misuse, of antibiotics, multidrug- or extensively-drug resistant (MDR/XDR)-bacteria have rapidly risen, generating a global health crisis affecting both human and animal health [3,4]. In this context, the search for new antimicrobial compounds has become imperative. Several approaches are under investigation, such as the use of enzymes, probiotics, antimicrobial peptides or bacteriophages, to name a few. Among these options, the Host Defense Peptides (HDPs) or antimicrobial peptides from innate immunity have stood out over others due to their natural versatility [5,6,7]. HDPs are short peptides, ranging from 12 to 50 amino acids, with cationic and amphiphilic features with a ubiquitous presence in nearly all biological kingdoms [8,9]. These evolutionary conserved molecules have an essential role in the innate immune system, regulating a broad range of immunological responses and modulating several biological signaling pathways related to processes such as wound healing, autoimmune disease and others [10,11]. Likewise, HDPs exhibit broad-spectrum activity against viruses, fungi and bacteria, including MDR/XDR strains [1,12] in both planktonic and biofilm forms since they can interact with the cell membrane through permeabilization or other antimicrobial mechanisms [12,13,14]. Resistance to HDPs could occur through several mechanisms, including changes in the bacterial cell wall or membrane or enzymatic degradation of the peptides, but research has shown that the risk of resistance to HDPs is lower than that of traditional antibiotics, in part because HDPs have a reduced half-life, which, combined with their variety of mechanisms of action, hamper the emergence of new resistances [15]. These peptides are broadly expressed in neutrophils and macrophages, being released during inflammatory responses, where they can act to either directly resolve infections by killing pathogenic bacteria or by coordinating immune responses [13,16].

**Figure 1:**
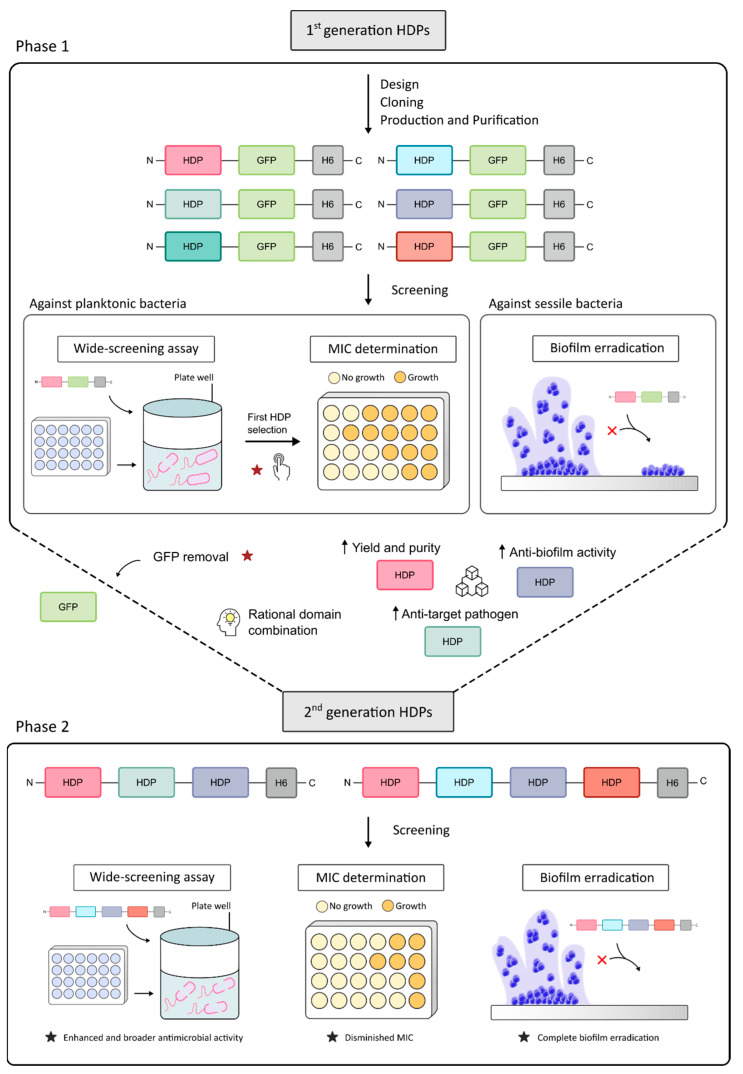
Scheme depicting the fabrication approach of enhanced broad-spectrum antimicrobials. The 1st generation of HDPs linked to a GFP carrier were evaluated in a triple assay, allowing the selection of the most promising ones to generate in phase 2, the 2nd generation of antimicrobials, devoid of a non-functional carrier, fully tunable and with enhanced antimicrobial features.

HDP production has commonly been carried out by chemical synthesis, although recombinant production has already been proven to be an alternative that allows producing these peptides through a scalable and cost-effective process, without limits in peptide length [17,18]. However, when produced in recombinant hosts, HDPs need to be fused to a carrier protein [19] to protect the peptide from host proteases and mask their possible toxic effect on the producer cell [17]. The removal of the carrier protein involves extra steps in the downstream purification and hence there is yield reduction and additional costs [20]. In this scenario, a recent study carried out by Roca-Pinilla et al. demonstrated that the combination of different functional HDP-based domains in a single polypeptide enabled the synthesis of a potent antimicrobial protein without compromising recombinant host viability and without the need of using protein carriers [21]. The present study aims to move a step forward through the development of a new generation of tailored antimicrobials using a rational design of multidomain proteins. This strategy is founded on a two-phase process (Figure 1), starting on the first generation of molecules produced from a library of HDPs fused to the carrier fluorescent protein Green Fluorescent Protein (GFP). After their testing against planktonic and biofilm forms of target pathogens, the best performing HDPs are combined in the second generation of chimeric molecules, where GFP is removed and tactical linkers are included, obtaining highly active and synergic HDP-based multidomain antimicrobial polypeptides.

## 2. Methods

### 2.1. Bacterial Strains 

*Escherichia coli* BL21 (DE3) was used for recombinant protein expression. To evaluate antimicrobial activity, the strains selected were methicillin-sensitive *Staphylococcus aureus* (MSSA, ATCC-3556), methicillin-resistant *Staphylococcus aureus* (MRSA, ATCC-33592), methicillin-resistant *Staphylococcus epidermidis* (MRSE, ATCC-35984) and *Pseudomonas aeruginosa* (ATCC-10145). *E. coli* strains were grown in Luria–Bertani (LB) medium, and MRSA, MSSA, MRSE and *P. aeruginosa* were grown in Brain Heart Infusion (BHI) broth (Scharlau, Barcelona, Spain). 

### 2.2. Genetic Construct Design

The 1st generation of molecules was based on the mature sequences of lingual antimicrobial peptide (LAP, Uniprot entry Q28880, V25-K64), human β-defensin 2 (HβD2, Uniprot entry O15263, G24-P64), human β-defensin 3 (HβD3, Uniprot entry P81534, G23-K67), human α-defensin 5 (HD5, Uniprot entry Q01523, A63-R94) and cathelicidin LL-37 (Uniprot entry P49913, L134-S170), fused to the GFP gene through the linker sequence GGSSRSS. The gene for the 2nd generation construct D5L37D5L37 comprised the combination of the repeated HD5 and LL-37 motifs, forming the HD5-LL-37-HD5-LL-37 construct. The gene encoding for D5LAL37βD3 consisted of the HD5, LAP, LL-37 and HβD3 sequences, and the D5L37βD3 construct was identical to D5LAL37βD3 removing the LAP domain. The same linker sequence was used to connect domain–domain sequences in 2nd generation molecules but removing the GFP gene. All constructs were C-terminally fused to a 6 histidine (H6)-tag for protein purification and a cysteine. The sequences were codon-optimized for the *E.* coli platform by GeneArt (GeneArt^®^, Life technologies, Regensburg, Germany), cloned into pET22b (*amp^R^*) and transformed by heat shock in competent *E. coli* BL21 (DE3) cells. Sequences of proteins are included in supplementary materials (Table S1).

### 2.3. Protein Production and Purification

Protein production was performed as previously described [22] and purification was performed from soluble fraction of recombinant cultures in all cases except proteins D5L37βD3 and D5LAL37βD3, which were obtained using inclusion bodies solubilization following established protocols [22].

### 2.4. Evaluation of Antimicrobial Activity

A triple antimicrobial assay was performed combining a broad screening antimicrobial assay, minimal inhibitory concentration (MIC) analyses and biofilm eradication (determination of Minimum Biofilm Eradication Concentration (MBEC)). Antibiotic MIC determination was included as a control (Figure S2). The broad screening antimicrobial assay was based on the BacTiter-Glo ^TM^ Microbial Cell Viability assay (Promega).Briefly s, an overnight (O/N) culture of the selected strain (MRSA, MSSA, MRSE or *P. aeruginosa*) was reinoculated in 10 mL of fresh BHI broth and grown at 250 rpm and 37 °C until an exponential growth phase was reached (OD_600_ = 0.4–0.6). Then, 150 μL from the bacterial diluted stock (10^6^–10^4^ cfu/mL) was centrifuged at 6200× *g* at 4 °C for 15 min. The supernatant was removed, and the bacterial pellet was resuspended with 150 µL of either 0.01% acetic acid (negative control) or 5 µM of antimicrobial protein treatment in 0.01% acetic acid and disposed in a sterile polypropylene 96-well plate (Costar). The 5 µM concentration was previosly determined with a compilation of previous exploratory experiments (Figure S1). After sample incubation for 5 h at 37 °C, 100 µL were withdrawn and tested with 100 µL of the BacTiter-Glo^TM^ reagent following the manufacturer’s instructions.

MIC was determined following performance standards for antimicrobial susceptibility testing with slight modifications [23]. Specifically, 10% Mueller Hinton Broth cation-adjusted medium (MHB-II, Sigma-Aldrich, St. Louis, MO, USA) was used to be more appropriate for testing cationic peptides and proteins [24]. Another relevant modification was the use of BacTiter-Glo^TM^ reagent to detect bacterial growth instead of visual observation since the growth was less evident because of the use of the diluted medium. Antibiofilm activity of each antimicrobial construct was assessed on pre-formed MRSA biofilms following the methodology described by Hancock et al. [25].

### 2.5. Protein Cytotoxicity Assay

Human peripheral blood mononuclear cells (PBMCs) were isolated from buffy coats of adult donors by density gradient centrifugation using Ficoll (Stemcell Technologies, Vancouver, BC, Canada). The buffy coats were provided by “Banc de Sang i Teixits de Barcelona (Spain)” under the approval No. 5099 of the “Ethics Committee on Animal and Human Experimentation” of the Autonomous University of Barcelona. The isolated PBMCs were plated in 96-well plates at a density of 2·10^5^ in 200 µL of RPMI-1640 medium supplemented with 10% fetal bovine serum (FBS) and 1% penicillin/streptomycin. The PBMCs were treated for 24 h with several concentrations of D5L37D5L37 in Hepes 20 mM. Then, PBMCs were stained with 0.5 μL of propidium iodide (Merck) during 3 min at room temperature before performing the flow cytometry measurements that were carried out in a CytoFLEX LX U3-V5-B3-Y5-R3-I0 Flow Cytometer (Beckman Coulter, Indianapolis, IN, USA).

### 2.6. SEM Imaging of Antimicrobial Effects 

Ultrastructural effects of 1st and 2nd generation constructs were assessed in *P. aeruginosa* and MRSA cultures. Briefly after, an O/N culture of both strains was 100-fold diluted in 10 mM KPi buffer. Then, 500 µL from the diluted bacteria was aliquoted and centrifuged at 6200× *g* and 4 °C for 15 min. The supernatant was removed, and the bacterial pellet was resuspended with 500 µL of antimicrobial construct at 5 µM in 0.01 % acetic acid. The treatments were disposed over coverglasses in a sterile 24-well plate and incubated for 5 min at 37 °C without agitation. After that, the supernatant was withdrawn, and the samples were fixed with 500 µL of 2.5% (*v*/*v*) glutaraldehyde (Merck, Rahway, NJ, USA) in 100 mM of phosphate buffer for 2 h at 4 °C. Following this, the coverglasses were washed with 100 mM phosphate buffer and fixed with 1% (*w*/*v*) osmium tetroxide-potassium ferrocyanide for 2 h. The samples were washed with miliQ water, dehydrated in a graded series of ethanol (50, 70, 90, 96 and 100% *v*/*v*) at RT and desiccated with hexamethyldisilazane (HMDS). Before microscopy observation, samples were metal-coated and then observed in a FESEM Merlin (Zeiss, Jena, Germany) operating at 3 kV.

### 2.7. Dynamic Light Scattering Measurements

The volume size distribution of 1st and 2nd generation molecules was determined in a Zetasizer Pro (Malvern Instruments Ltd., Malvern, UK) by dynamic light scattering (DLS). The protein concentration was adjusted to 1 mg/mL in 0.01 % acetic acid buffer, at pH 3.8. A 100 µL aliquot (stored at −80 °C) was thawed and then centrifuged at 15,000× *g* for 15 min at 4 °C to remove non-specific aggregates. Further, the supernatants were measured in triplicate, and the average size and polydispersity index (PI) were displayed as mean ± SEM.

### 2.8. Statistical Analysis

Results are expressed as the means of non-transformed data ± standard error of the mean (SEM). Data were obtained in triplicate, and normality was checked using JMP software (SAS Institute Inc.), being transformed when required. The *p*-values (statistically significant when *p* < 0.05) and letters correspond to the ANOVA and Tukey test analyses.

## 3. Results

### 3.1. First Generation of HDP-Based Antimicrobial Proteins

**Figure 2:**
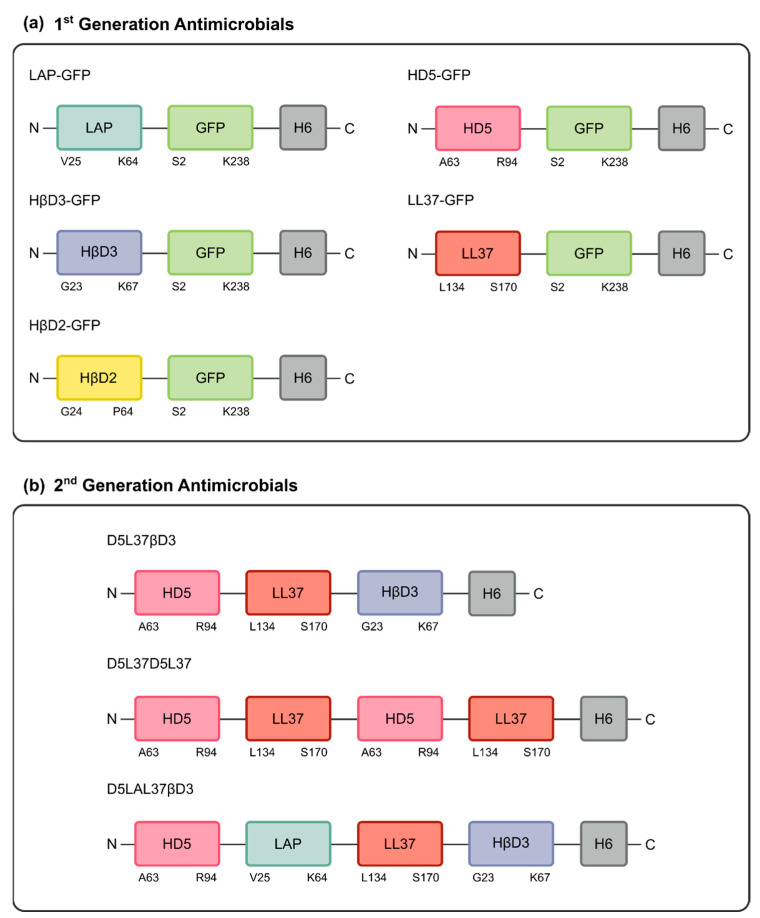
Schematic representation of both 1st and 2nd generations of antimicrobial proteins. (**a**) The 1st generation constructs are constituted from N- to C-terminal by a single HDP-based domain (LAP, HβD2, HβD3, HD5 or LL37) fused to the GFP gene. (**b**) The 2nd generation constructs are multidomain proteins combining HD5, LL37 and HβD3 domains (D5L37βD3), combining the last three with LAP (D5LAL37βD3) and using HD5 and LL37 tandem repetitions (D5L37D5L37). All constructs have an H6-tag at C-terminal for protein purification purposes.

The first generation of antimicrobials was obtained by fusing the codifying region of five different HDPs to the GFP gene and a H6-tag (Figure 2a) and they were successfully produced in the soluble fraction of recombinant *E. coli*. After IMAC purification, good yields and purity were achieved for all proteins (Table 1).

**Figure 3:**
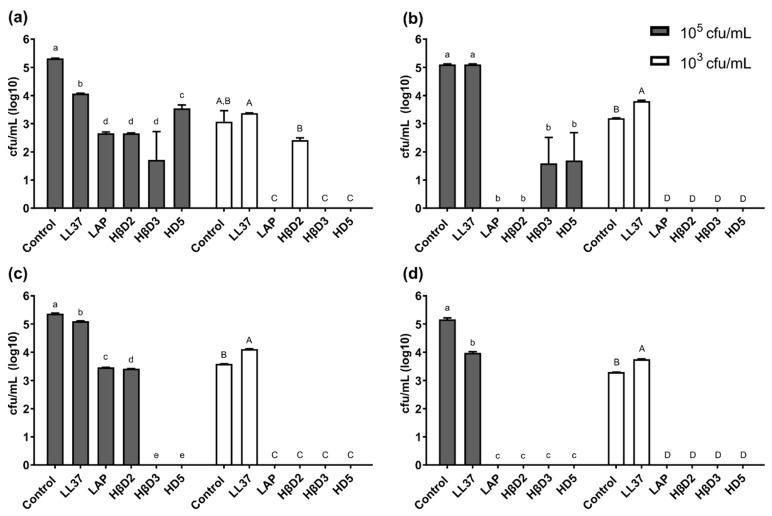
Antimicrobial activity of 1st generation antimicrobials. Antimicrobial activity (reduction of log of cfu/mL) of the different 1st generation constructs against (**a**) MRSA, (**b**) MSSA, (**c**) MRSE and (**d**) *P. aeruginosa*. The constructs were tested against an initial concentration of 10^5^ cfu/mL (dark bars) or 10^3^ cfu/mL (white bars). Data shown are the mean of a triplicate ± SEM. Different letters depict statistically significant differences (*p* < 0.0001) examined by ANOVA and Tukey test analysis. Capital and lowercase letters refer to 10^5^ cfu/mL and 10^3^ cfu/mL, respectively.

The antimicrobial activity of first generation molecules was evaluated against both Gram-positive MSSA, MRSA and MRSE and the Gram-negative bacteria *P. aeruginosa.* The testing of antimicrobial activity was carried out in three steps: (1) wide screening antimicrobial assay, (2) determination of the MIC of HDPs selected in the first step and (3) biofilm eradication and MBEC determination (Figure 1). The wide screening assay showed that most active molecules were those based on HβD3 and HD5, reducing at least 3-log in all bacterial pathogens (Figure 3) and 5-log in MRSE *and P. aeruginosa* (Figure 3c,d). The LAP and HβD2-based protein activity were strain-dependent, killing completely MSSA and *P. aeruginosa,* either at 10^5^ and 10^3^ cfu/mL (Figure 3b,d, respectively), but showing lower performance against the MRSA and MRSE strains (Figure 3a,c, respectively). On the other hand, the LL-37-based construct only showed mild bactericidal effects against MRSA (Figure 3a), MRSE (Figure 3c) and *P. aeruginosa* (Figure 3d) at 10^5^ cfu/mL and was not selected in the next step of the MIC determination.

**Figure 4:**
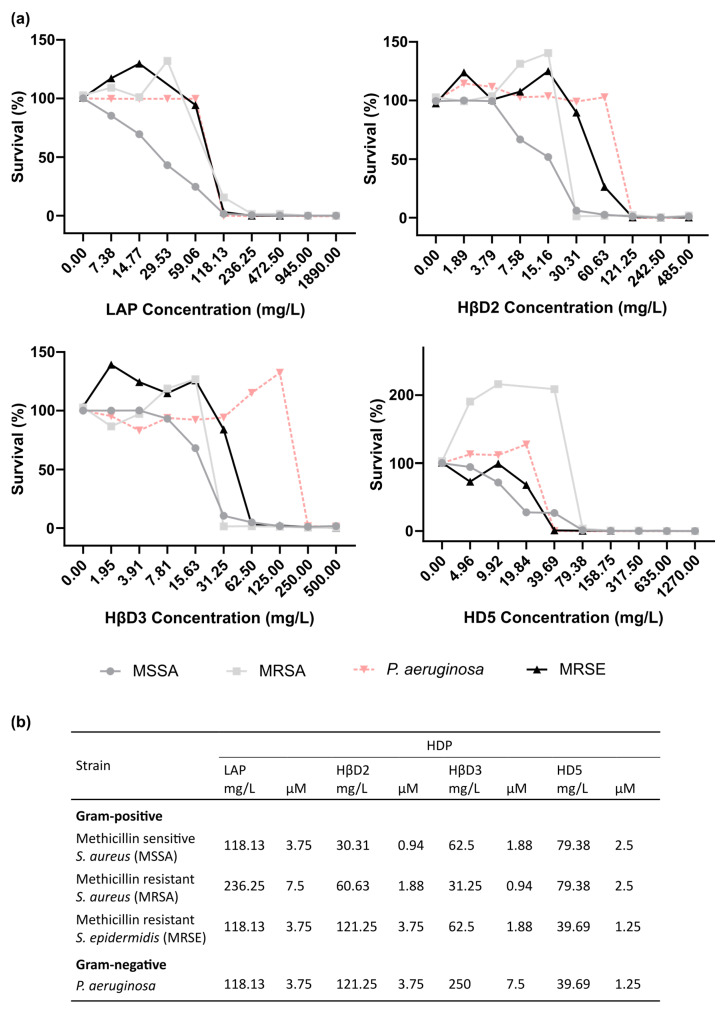
Minimal inhibitory concentration of 1st generation antimicrobials. (**a**) Minimal inhibitory concentration (MIC) assay of proteins based on LAP, HβD2, HβD3 and HD5 against MRSA (filled squares), MSSA (filled circles), MRSE (filled triangles) and *P. aeruginosa* (filled inversed triangles). Each construct was tested at its maximum concentration and serial two-fold dilution to determine MIC against the four tested microorganisms. (**b**) Summary of MIC values.

The LAP-based construct had an MIC ranging from 236.25 mg/L against MRSA to 118.13 mg/L against MSSA, MRSE and *P. aeruginosa* (Figure 4). HβD2 showed the same MIC value (121.25 mg/L) for Gram-negative *P. aeruginosa* and Gram-positive MRSE. However, the MIC was much better against Gram-positive MRSA and MSSA, being 60.63 mg/L and 30.31 mg/L, respectively. The HβD3-based protein had a high MIC of 250 mg/L for *P. aeruginosa,* but it decreased considerably against Gram-positive MRSA, MSSA and MRSE, being 62.5 mg/L, 31.25 mg/L and 62.50 mg/L, respectively. Finally, the HD5 construct showed a similar performance against Gram-positive and Gram-negative strains, with MIC values between 79.38 mg/L against MRSA and MSSA and 39.96 mg/L against MRSE and *P. aeruginosa* (Figure 4b).

**Figure 5:**
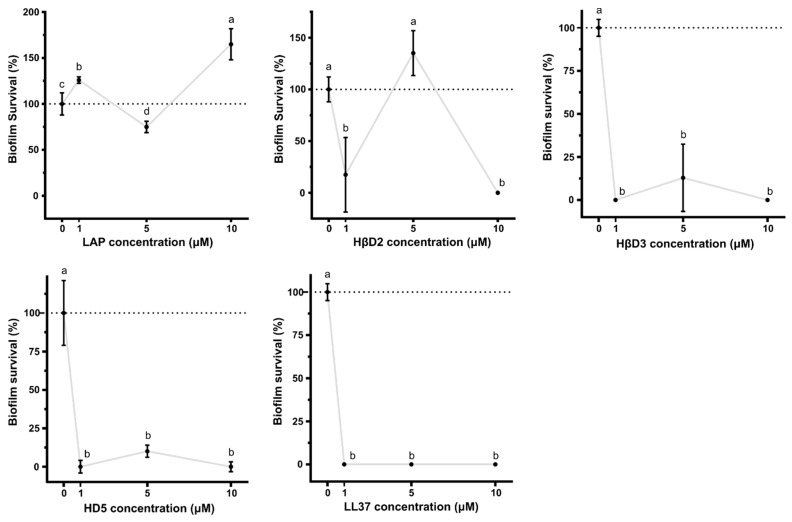
Antibiofilm performance of 1st generation molecules. Antibiofilm activity of the different 1st generation constructs at 10, 5 and 1 µM, against pre-formed biofilm of MRSA. Complete survival of biofilm (100%) is indicated by dotted lines. Data shown are the mean of triplicate ± SEM. Different letters represent statistically significant differences (*p* < 0.0001) assessed by ANOVA and Tukey test analysis.

**Figure 6:**
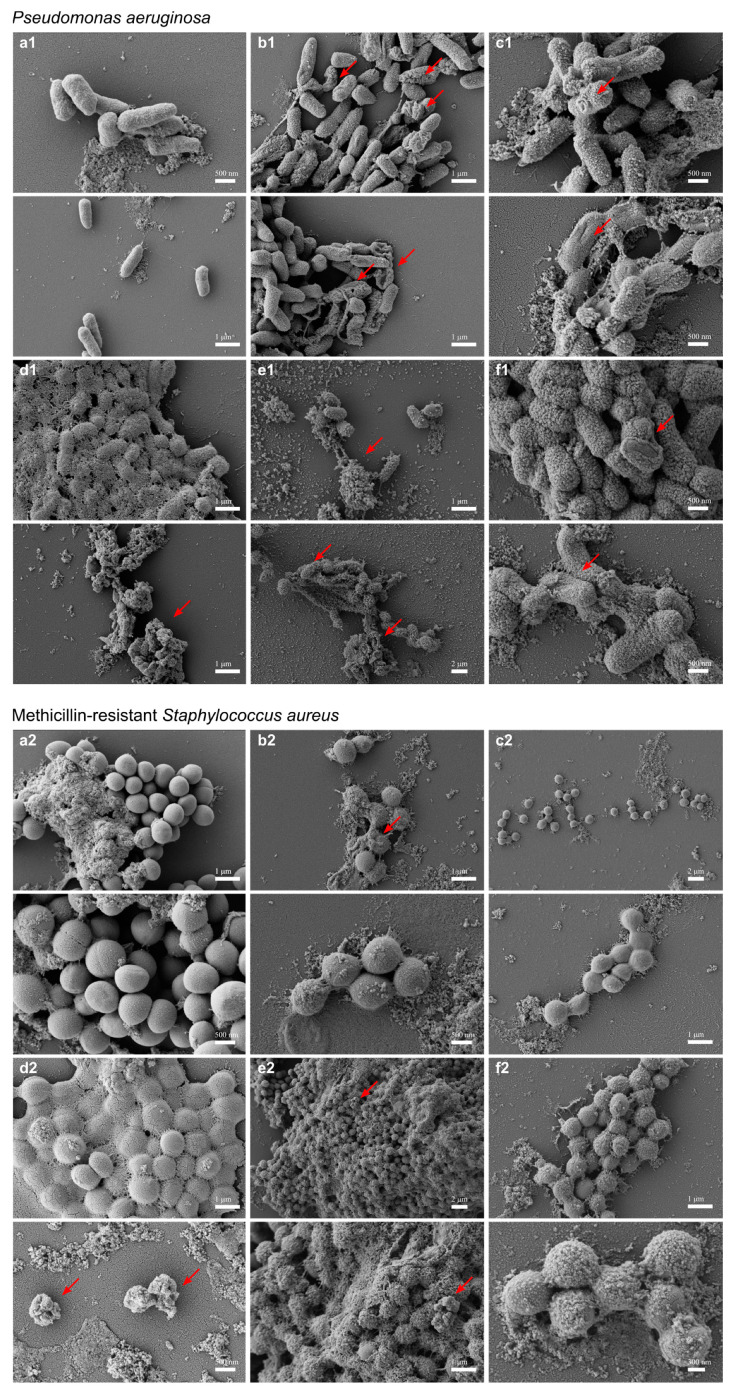
Analysis of the antimicrobial mechanisms using scanning electron microscopic study (FE-SEM). FESEM cell integrity images of *P. aeruginosa* and MRSA after (**a**) control, (**b**) HD5, (**c**) HβD3, (**d**) LL-37 1st generation construct treatment, (**e**) D5L37D5L37 and (**f**) D5L37βD3 multidomain proteins (2nd generation antimicrobial) treatment. All treatments were applied at 5 µM. Scale bars are indicated in each image. Red arrows point out relevant image areas.

HβD3, HD5 and LL-37-based proteins exhibited strong antibiofilm features in a dose-independent manner, reducing the biofilm survival almost by 100% in the three tested concentrations (*p* < 0.0001) (Figure 5). The MBEC obtained for HβD2-based protein was dose-variable since it worked at 1 and 10 µM but not at 5 µM. Surprisingly, LAP-based protein was not able to reduce biofilm formation. Finally, the morphological changes in *P. aeruginosa* and MRSA were assessed by electron microscopy after 5 min of incubation with first generation constructs. The bacteria controls without HDP-based proteins (Figure 6a1,a2) exhibited smooth surfaces, but those incubated with first generation antimicrobials appeared to clump and showed crenated surfaces for both HD5 and HβD3 (Figure 6b1,b2,c1,c2) along with the presence of sparse pores in the case of *P. aeruginosa* (Figure 6b1). However, for the LL-37 treatment, cells appeared to be clumped and embedded in a whole cell debris and a mucus-like layer (Figure 6d1,d2).

### 3.2. Second Generation of HDP-Based Antimicrobial Proteins

**Figure 7:**
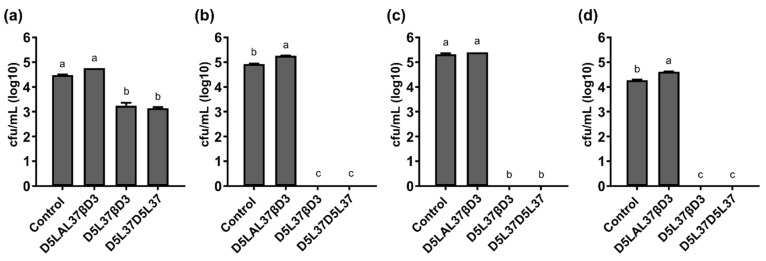
Bactericidal activity of 2nd generation HDPs. Antimicrobial activity of D5L37D5L37, D5L37βD3 and D5LAL37βD3 multidomain constructs at 5 μM against (**a**) MRSA, (**b**) MSSA, (**c**) MRSE and (**d**) *P. aeruginosa.* All constructs were tested against an initial 10^5^ cfu/mL of each of the bacteria. Data shown are the mean of triplicate ± SEM. Different letters represent significant differences (*p* < 0.0001) assessed by ANOVA and Tukey test analysis.

After the functional evaluation of first generation proteins, and considering the production yield of each protein, HD5, LAP, LL-37 and HβD3 were selected for the modular protein design of the second generation of antimicrobial proteins (Figure 1). As a first proof of concept, three proteins were constructed (Figure 2b) and produced in *E. coli* at good purity levels but at lower yields than the first generation proteins (Table 2). Proteins D5L37βD3 and D5L37D5L37 reduced 1.5-log the bacterial load of MRSA (Figure 7a) and 5-log in total that of MSSA (Figure 7b), MRSE (Figure 7c) and *P. aeruginosa* (Figure 7d) (*p* < 0.0001). However, the construct D5LAL37βD3 did not show antimicrobial activity against the planktonic form of any of the four tested pathogens (Figure 7).

**Figure 8:**
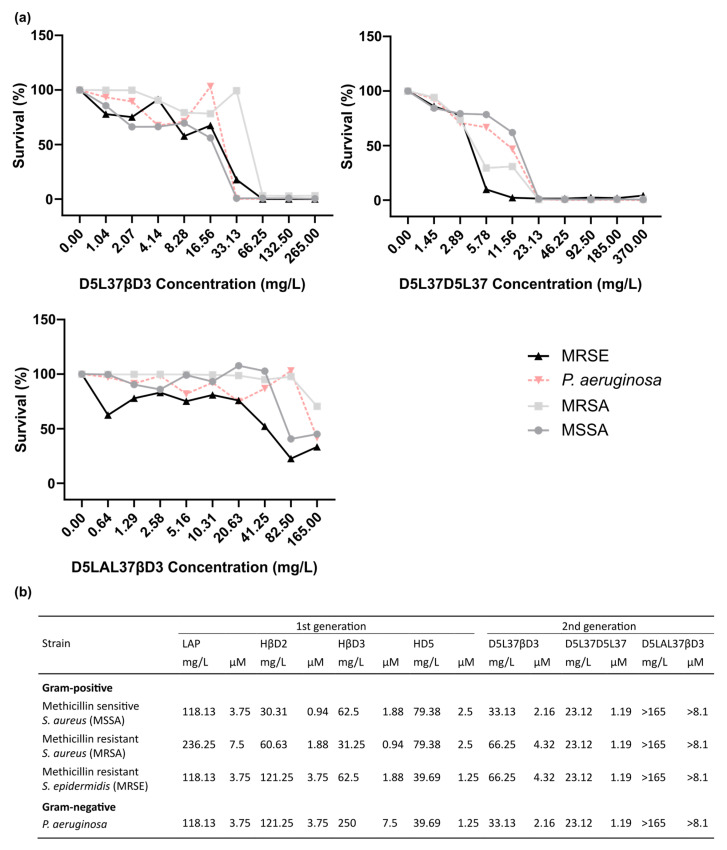
Optimized minimal inhibitory concentration of multidomain antimicrobial proteins. (**a**) MIC of the 2nd generation of antimicrobial constructs D5L37βD3, D5L37D5L37 and D5LAL37βD3 against MRSA (filled squares), MSSA (filled circles), MRSE (filled triangles) and *P. aeruginosa* (filled inversed triangles). All constructs were evaluated at their maximum achieved concentration and a serial of two-fold dilution was performed to determine MIC against the examined microorganism. (**b**) Summary of MIC values of 1st and 2nd generation.

**Figure 9:**
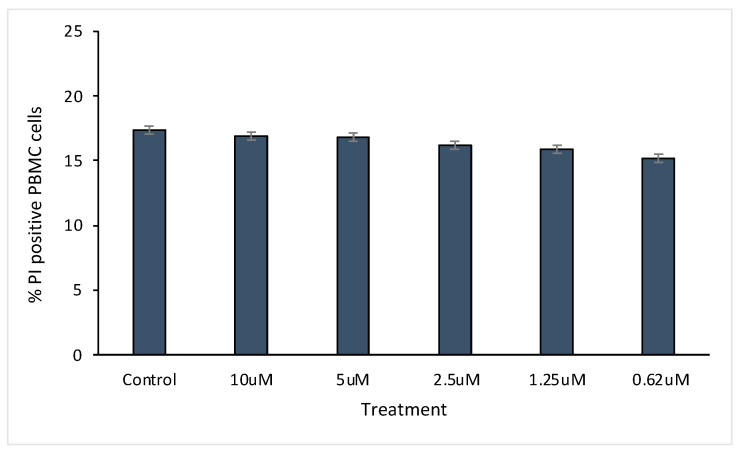
Protein toxicity assay. Percentage of propidium iodide positive PBMC as a measure of toxicity after 24 h treatment with several concentrations of protein D5L37D5L37.

**Figure 10:**
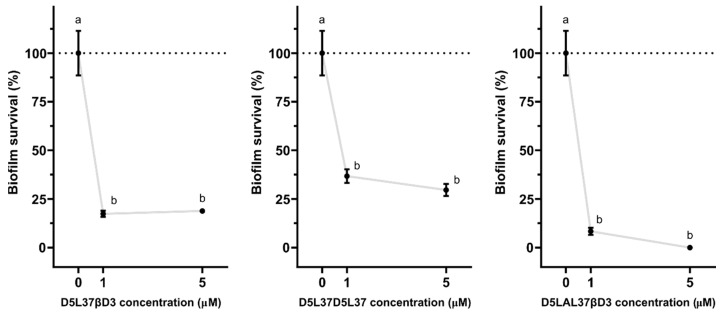
Antibiofilm performance of 2nd generation molecules. Biofilm eradication capacity of the different multidomain constructs D5L37βD3, D5L37D5L37 and D5LAL37βD3 at 5 and 1 μM against MRSA pre-formed biofilms. Complete survival of biofilm (100%) is indicated by dotted lines. Plots are the mean of triplicate ± SEM. Different letters indicate statistically significant differences (*p* < 0.0001) assessed by ANOVA and Tukey test analysis.

The MIC values using D5L37D5L37 (Figure 8b) were the lowest achieved, being 26.88 mg/L for all tested organisms (MRSA, MSSA, *S. epidermidis* and *P. aeruginosa*) but it did not show any signs of cytotoxicity in PBMC cells (Figure 9). The D5L37βD3 construct inhibited the growth of MRSE and *P. aeruginosa* with an MIC of 31.25 mg/L in both cases and 62.50 mg/L for MRSA and MSSA (Figure 8b). The MIC for the D5LAL37βD3 construct was greater than the maximum concentration that could be tested (Figure 8a) and it was not possible to be determined. Additionally, the antimicrobial activity of second generation proteins was evaluated in biofilm eradication, and the three proteins had an MBEC between 1 and 5 µM (Figure 10). The best biofilm inhibition rates (almost 100%) were achieved with D5LAL37βD3.

The morphological evaluation of *P. aeruginosa* and *MRSA* were analyzed using electron microscopy after incubation with second generation constructs. The non-treated bacteria (Figure 6a1,a2) exhibited smooth surfaces, in contrast with bacteria incubated with antimicrobials, which exhibited rough and micelle-like surfaces for both D5L37D5L37 and D5L37βD3 multidomain proteins (Figure 6e1,e2,f1,f2).

### 3.3. Physicochemical Characterization of First and Second Generation of Antimicrobials

**Figure 11:**
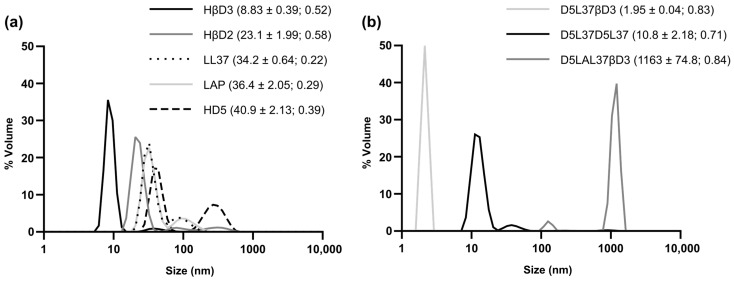
Characterization of recombinant HDPs structuration. Size distribution plots of (**a**) 1st generation proteins based on HβD2, HβD3, LL37, LAP and HD5, and (**b**) 2nd generation proteins D5LAL37βD3, D5L37D5L37 and D5L37βD3. The mean size ± SEM and polydispersity index (PI) are indicated in brackets.

The mean hydrodynamic particle size of HDP-based proteins from both generations was assessed by DLS (Figure 11a,b). The HβD3-based construct exhibited a predominant peak at 8.83 nm, whereas the HβD2, LAP, LL37 and HD5 showed a larger particle size, varying from 23.1 to 40.9 nm (Figure 11a). The LL37, LAP and HD5-based construct profiles also pointed out the existence of multiple populations in a dynamic equilibrium, generating the appearance of multiple peaks instead of one (Figure 11a). The second generation molecules presented heterogeneous profiles among them (Figure 11b), where peaks ranged from 1.95 nm for D5L37βD3 to 1163 nm in the case of D5LAL37βD3. The protein D5L37D5L37 showed a predominant peak of 10.8 nm, similar to those found for the first generation molecules.

## 4. Discussion

The results obtained herein proved that the first and second generation antimicrobials were successfully produced. The recombinant host toxicity was reduced in the first generation proteins, probably because of the carrier protein (GFP) presence, which is in agreement with the use of other function-related carriers, such as the thioredoxin, glutathione-S-transferase or the small ubiquitin-related modifier (SUMO), compensating the HDP sequence [26]. However, despite the lower yields of the second generation proteins, they were good enough to be produced and purified at reasonable and scalable levels. Altogether, this proved that a two-phase procedure was worthy to take advantage of a carrier protein for a wide screening of HDPs against target pathogens to design multidomain proteins combining the most promising ones.

The antimicrobial activity obtained with the first generation molecules, except for HβD3, was not dependent on Gram-positive or Gram-negative microorganisms but had a pathogen-specific effect (Figure 3 and Figure 4). The same profile was also confirmed in the second generation of molecules. HβD3 showed a preferred antimicrobial activity against the Gram-positive MRSA, MSSA and *S. epidermidis*, in contrast to Gram-negative *P. aeruginosa* (Figure 4b). These differences in performance might be explained by different structural bacterial wall compositions between Gram-positive and Gram-negative bacteria. However, the rest of the HDPs were strain-specific, probably because, although the main mechanism of cell death is based on membrane disruption, the HDPs can also penetrate the bacterial cell wall and interfere with a vast array of intracellular targets [27], inhibiting DNA replication or bacterial protein synthesis, leading to cell death.

Herein, the functional selection of antimicrobials is based on three complementary assays. In the first wide screening assay, two initial culture concentrations of bacteria (10^5^ and 10^3^ cfu/mL) were used, and we concluded that 10^5^ cfu/mL was the optimal one to finely evaluate the antimicrobial potential (Figure 3) since the protein activity could be overestimated when working at 10^3^ cfu/mL. The first wide screening allowed us to discard proteins with low efficiency against the planktonic cultures, not being necessary to be purified at high amounts to perform the MIC assay. Thus, the MIC of all the first generation molecules was determined for all proteins, except LL-37 (Figure 4). The MIC assay determines the minimal concentration of an antimicrobial necessary to inhibit bacterial growth, while the first wide assay enables evaluation of the plain bactericidal activity at 5 µM. HDPs with similar activities in wide screening assays (Figure 3) showed clear differences in MIC values (Figure 4b), proving that this analysis is a complementary tool to evaluate antimicrobial capacity. The last activity assay was the biofilm eradication or MBEC determination, where all the proteins were tested independently of the results obtained with planktonic cells (Figure 5). To perform this analysis, MRSA was chosen as an indicator strain because it was the most consistent bacteria forming biofilms within all four pathogens. Bacteria embedded in a biofilm undergo several phenotypic modifications. This condition hampers bacterial killing because of the slow bacterial growth and presence of an extracellular matrix that avoids antimicrobial compound diffusion. In accordance with this, despite the significant antimicrobial activity shown by LAP against planktonic bacteria (Figure 3 and Figure 4), it was not effective against biofilms (Figure 5). On the contrary, HβD3 and HD5-based proteins selected previously for their good activity in planktonic cultures (Figure 3 and Figure 4) had also good activity against biofilms (Figure 5). Finally, the LL-37 protein, with a bad performance against planktonic cultures, was the best candidate against biofilms of MRSA. This difference in LL-37 performance could be attributed to its well-known activity affecting the quorum sensing of the biofilm [13]. In fact, the electron microscopy images showed that LL-37 performed differently to other HDPs since the morphological aspect of treated bacterial cells was surprisingly different (Figure 6). The images suggested that LL-37 was able to affect the whole culture at once but not from a single cell point of view.

Considering the results obtained from the triple activity assay for the first generation proteins (Figure 1 Phase 1), we selected HDPs for the second generation construction following criteria of best antimicrobial performance and recombinant yield. The first selected domains were HβD3 and HD5 due to their potent antimicrobial (Figure 3, Figure 4 and Figure 8b) and antibiofilm activities (Figure 5). Secondly, LAP, which had also a good performance against planktonic bacteria (Figure 3, Figure 4 and Figure 8b) and elevated production yields (Table 1), was also selected. Finally, LL37, which exhibited the strongest antibiofilm properties (Figure 5), was also chosen. Combining these HDPs in a random position, we evaluated three plausible multidomain candidates. Still, the potentiality of this approach allows us to structure a myriad of combinations, improving the performance in a rational strategy (Figure 1 Phase 2). In fact, we still do not know the impact of combining HDPs with distinct modes of action in a single polypeptide (for instance, HD5 operates through a pore-forming mechanism, while HβD3 and LL-37 act in a carpet-like fashion). The rules behind the domain order and the number of domains used must be studied in further studies combining biocomputational and experimental approaches. Herein, the used domain combination triggered a synergistic effect, which is directly reflected in an enhanced bactericidal activity (Figure 7). In fact, the HD5 construct of the first generation was only able to reduce 3-log the bacterial survival of MSSA (Figure 3), whereas the D5L37D5L37 construct showed a 5-log reduction in this strain (Figure 7). In general, both D5L37D5L37 and D5L37βD3 exhibited a high antimicrobial performance against MSSA, MRSE and *P. aeruginosa*, whereas the MRSA strain was more resistant to the treatment (Figure 7). This activity improvement can be also observed in the MIC assay, where the values ranged from 62.50 to 26.88 mg/L (Figure 8b). Remarkably, the construct D5L37D5L37 exhibited the lowest MIC values, indicating that the role of domain repetitions in antimicrobial performance must be further evaluated extensively. The second generation D5L37D5L37 protein was the best broad-spectrum antimicrobial selected, presenting MICs of 23,12 mg/L (1.19 µM) against all pathogens and without signs of toxicity on PBMC. These MICs were better than those obtained with the best performing hybrid synthetic peptides already published, which were 2 µM and 4 µM for *P. aeruginosa* and *S. aureus*, respectively [28]. Surprisingly, the D5LAL37βD3 construct did not show any antimicrobial activity against planktonic bacteria (Figure 7 and Figure 8), although it showed good antibiofilm activity (Figure 10). This probably indicates an incorrect domain structure, folding or accessibility due to the large assembly detected by DLS for this protein sample (Figure 11b). In addition, the DLS peaks indicated that the first generation proteins might be structured in dimers or oligomers (Figure 11). In fact, this has been described in modular recombinant proteins with a configuration of the cationic peptide-GFP-H6, the formation of protein nanoparticles favored by intermolecular interactions of monomers [29]. In agreement with this, the DLS analysis of the first generation of the constructs HDP-GFP-H6 showed the presence of discrete populations of conformers between 10 and 40 nm (Figure 11a). Furthermore, the DLS analysis of the second generation protein D5L37D5L37 identified a main population of 10.8 nm which had a remarkable performance in both planktonic and biofilm challenges. As recombinant proteins are unstable molecules, the formation of protein nanoparticles may give an additional advantage in administration routines and merits further studies. Surprisingly, even though the D5L37βD3 construct was detected as an unassembled form, its antimicrobial activity was also noticeable. On the other hand, the DLS analysis of D5LAL37βD3 revealed the presence of a 1163 nm peak, which indicated high molecular assemblies (aggregates) that possibly impair its activity against planktonic culture. However, this may offer a high and stable local concentration of HDP that favors the antibiofilm effect. Thus, in general terms, we can conclude that protein folding of each domain in the performance of the second generation molecules could have a potential impact on the final activity, this being a parameter that needs to be further explored.

In addition to their antibacterial properties, HDPs are known to have other functions, such as immunomodulatory effects, LPS neutralization and wound healing [30]. Further studies must assess whether these multifunctional properties are retained when the HDPs are incorporated into multidomain proteins to both open new applications and to explore side effects that must be modulated by targeting properties or specific combinations in the final multidomain proteins.

## 5. Conclusions

We have developed and proved a novel strategy to generate new broad-spectrum antimicrobials based on HDPs. Particularly, the D5L37D5L37 compound exhibited the best antimicrobial performance against the four human pathogenic-related strains, being a plausible candidate to be further investigated with other strains of pathogens tested.

## Figures and Tables

**Table 1:** Antimicrobial protein yield (mg L^−1^ culture) and purity (%) of soluble LAP, HβD2, HβD3, HD5 and LL37. ^a^ Yields calculated after protein purification.

Protein	Yield (mg/L) ^a^	Purity (%)
LAP	23.4	>99
HβD2	3.48	97
HβD3	1.84	89
HD5	5.84	>99
LL37	1.74	58

**Table 2:** Second generation antimicrobial protein yield (mg/L culture) and purity (%) of soluble D5L37βD3, D5L37D5L37 and D5LAL37βD3. ^a^ Yields calculated after protein purification.

Protein	Yield (mg/L) ^a^	Purity (%)
D5L37βD3	0.44	97
D5L37D5L37	0.11	87
D5LAL37βD3	0.15	86

## Data Availability

The data can be shared up on request.

## Supplementary Materials

The following supporting information can be downloaded at https://www.mdpi.com/article/10.3390/pharmaceutics15041068/s1. Figure S1: Dose-response determination of the 1st generation antimicrobials. Figure S2: Minimal inhibitory concentration of relevant antibiotics. Table S1: Sequences of 1st generation and 2nd generation molecules.
