# Supplementary material for: A Novel Generation of Tailored Antimicrobial Drugs Based on Recombinant Multidomain Proteins
Source: Pharmaceutics. 2023 Mar 26;15(4):1068. doi: 10.3390/pharmaceutics15041068 (PMC10146347; doi:10.3390/pharmaceutics15041068)
Supplement: Supplementary file 1 [file pharmaceutics-15-01068-s001.zip › pharmaceutics-2237628-supplementary.pdf]

Supplementary material

Table S1. Sequences of 1st generation and 2nd generation molecules. Each HDP is represented in one color and GFP is in light green. GGSSRSS is the linker sequence.

Protein	Sequence
LAP-GFP	MVRNSQSCRRNKGICVPIRCPGSMRQIGTCLGAQVKCCRRKGGSSRSSSKGEELFTGVVPILVELD GDVNGHKFSVSGEGEGDATYGKLTLKFICTTGKLPVPWPTLVTTLTYGVQCFSRYPDHMKRHDF FKSAMPEGYVQERTISFKDDGNYKTRAEVKFEGDTLVNRIELKGIDFKEDGNILGHKLEYNYNSH NVYITADKQKNGIKANFKIRHNIEDGSVQLADHYQQNTPIGDGPVLLPDNHYLSTQSALSKDPNE KRDHMVLLEFVTAAGITHGMDELYKHHHHHHC
HD5-GFP	MATCYCRTGRCATRESLSGVCEISGRLYRLCCRGGSSRSSSKGEELFTGVVPILVELDGDVNGHKF SVSGEGEGDATYGKLTLKFICTTGKLPVPWPTLVTTLTYGVQCFSRYPDHMKRHDFFKSAMPEG YVQERTISFKDDGNYKTRAEVKFEGDTLVNRIELKGIDFKEDGNILGHKLEYNYNSHNVYITADK QKNGIKANFKIRHNIEDGSVQLADHYQQNTPIGDGPVLLPDNHYLSTQSALSKDPNEKRDHMVLL EFVTAAGITHGMDELYKHHHHHHC
HβD3-GFP	MGIINTLQKYYCRVRGGRCAVLSCLPKEEQIGKCSTRGRKCCRRKKGGSSRSSSKGEELFTGVVPIL VELDGDVNGHKFSVSGEGEGDATYGKLTLKFICTTGKLPVPWPTLVTTLTYGVQCFSRYPDHMK RHDFFKSAMPEGYVQERTISFKDDGNYKTRAEVKFEGDTLVNRIELKGIDFKEDGNILGHKLEYN YNSHNVYITADKQKNGIKANFKIRHNIEDGSVQLADHYQQNTPIGDGPVLLPDNHYLSTQSALSK DPNEKRDHMVLLEFVTAAGITHGMDELYKHHHHHHC
HβD2-GFP	MGIGDPVTCLKSGAICHPVFCPRRYKQIGTCGLPGTKCCKKPGGSSRSSSKGEELFTGVVPILVELD GDVNGHKFSVSGEGEGDATYGKLTLKFICTTGKLPVPWPTLVTTLTYGVQCFSRYPDHMKRHDF FKSAMPEGYVQERTISFKDDGNYKTRAEVKFEGDTLVNRIELKGIDFKEDGNILGHKLEYNYNSH NVYITADKQKNGIKANFKIRHNIEDGSVQLADHYQQNTPIGDGPVLLPDNHYLSTQSALSKDPNE KRDHMVLLEFVTAAGITHGMDELYKHHHHHHC
LL37-GFP	MLLGDFFRKSKEKIGKEFKRIVQRIKDFLRNLVPRTESGGSSRSSSKGEELFTGVVPILVELDGDVN GHKFSVSGEGEGDATYGKLTLKFICTTGKLPVPWPTLVTTLTYGVQCFSRYPDHMKRHDFFKSA MPEGYVQERTISFKDDGNYKTRAEVKFEGDTLVNRIELKGIDFKEDGNILGHKLEYNYNSHNVYI TADKQKNGIKANFKIRHNIEDGSVQLADHYQQNTPIGDGPVLLPDNHYLSTQSALSKDPNEKRDH MVLLEFVTAAGITHGMDELYKHHHHHHC
D5L37βD3	MATCYCRTGRCATRESLSGVCEISGRLYRLCCRGGSSRSSLLGDFFRKSKEKIGKEFKRIVQRIKDF LRNLVPRTESGGSSRSSGIINTLQKYYCRVRGGRCAVLSCLPKEEQIGKCSTRGRKCCRRKKHHHH HHC
D5L37D5L37	MATCYCRTGRCATRESLSGVCEISGRLYRLCCRGGSSRSSLLGDFFRKSKEKIGKEFKRIVQRIKDF LRNLVPRTESGGSSRSSATCYCRTGRCATRESLSGVCEISGRLYRLCCRGGSSRSSLLGDFFRKSKE KIGKEFKRIVQRIKDFLRNLVPRTESHHHHHHC
D5LAL37βD3	MATCYCRTGRCATRESLSGVCEISGRLYRLCCRGGSSRSSVRNSQSCRRNKGICVPIRCPGSMRQI GTCLGAQVKCCRRKGGSSRSSLLGDFFRKSKEKIGKEFKRIVQRIKDFLRNLVPRTESGGSSRSSGII NTLQKYYCRVRGGRCAVLSCLPKEEQIGKCSTRGRKCCRRKKHHHHHHC

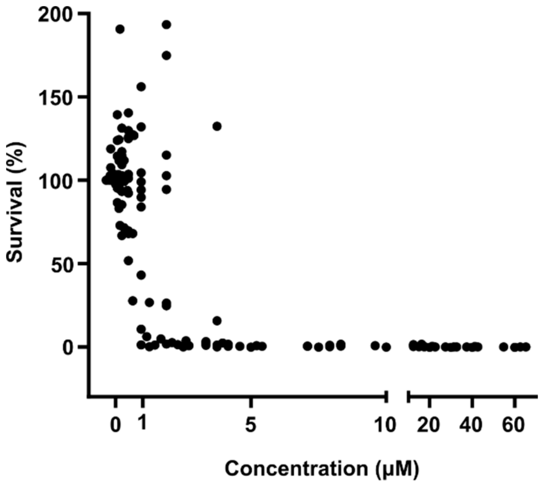

Figure S1. Dose-response determination of the 1st generation antimicrobials. Compilation raw data of all bactericidal assays performed in the laboratory with LAP, HβD2, HβD3 and HD5 against methicillin resistant *Staphylococcus aureus* (MRSA), methicillin sensitive *Staphylococcus aureus* (MSSA), methicillin resistant *Staphylococcus epidermidis* (MRSE) and *Pseudomonas aeruginosa*. Each point of the serial two-fold diluted antimicrobial concentration was illustrated to determine the optimal HDPs microbicidal concentration.

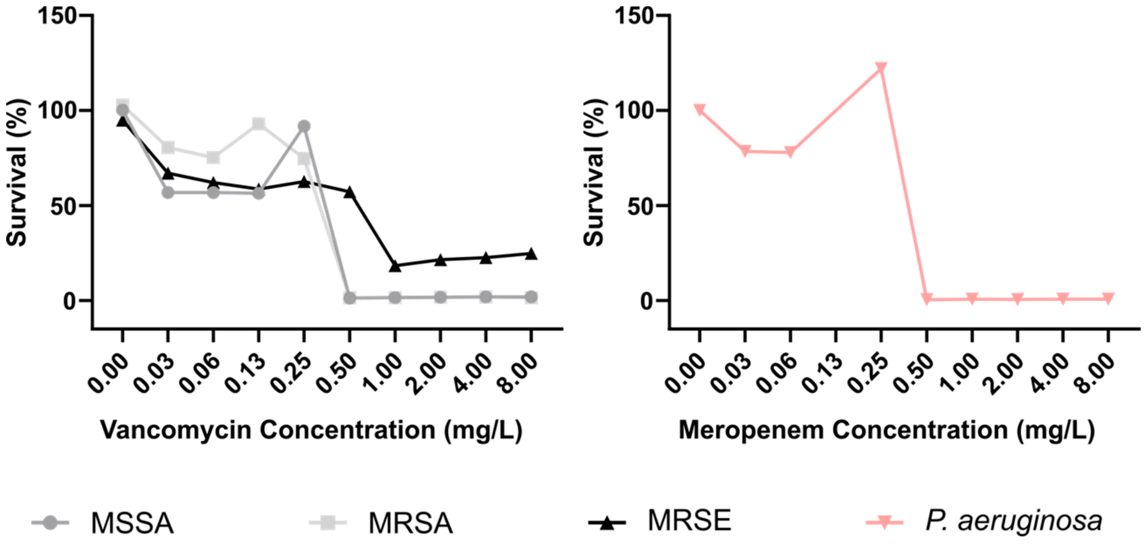

Figure S2. Minimal inhibitory concentration of relevant antibiotics. Minimal inhibitory concentration (MIC) assay of Vancomycin and Meropenem against methicillin-resistant *Staphylococcus aureus* (filled squares), methicillin-sensitive *Staphylococcus aureus* (filled circles), methicillin-resistant

Staphylococcus. epidermidis (filled triangles) and *Pseudomonas aeruginosa* (filled inversed triangles), respectively. Each antibiotic was tested in a serial two-fold dilution to determine MIC against the four tested microorganisms, validating the strategy proposed.
